# Understanding the Pathogenesis of Lateral Supratentorial Neurenteric Cysts in Close Proximity to Other Vascular Pathologies: A Case Report and Review of Embryology

**DOI:** 10.7759/cureus.25608

**Published:** 2022-06-02

**Authors:** Mohamed M Salem, Kyle Mccloskey, Dominic Romeo, Maria Gubbiotti, YouRong Sophie Su, Dennis M DePace, Brian T Jankowitz, Jan-Karl Burkhardt

**Affiliations:** 1 Neurosurgery, University of Pennsylvania Perelman School of Medicine, Philadelphia, USA; 2 Pathology, University of Pennsylvania Perelman School of Medicine, Philadelphia, USA; 3 Anatomy, Drexel University College of Medicine, Philadelphia, USA

**Keywords:** microsurgery, neurenteric cysts, aneurysm, cavernoma, embryology

## Abstract

Several theories have been postulated to explain the embryogenesis of central nervous system (CNS) neurenteric cysts (NCs), but the exact mechanism remains poorly understood. Of those, the neurenteric canal migration hypothesis suggesting endodermal cell migration through the neurenteric canal and settling among ectodermal cells prior to neural tube closure might be the most robust as it explains, in contrast to other hypotheses, the existence of lateral supratentorial lesions, which are extremely rare, compared to their infratentorial counterparts. This mechanism might be supported by past medical history or the coexistence of CNS epidermoid cysts, which are thought to arise due to improper neural tube closure potentially increasing the probability of endodermal migration and subsequent NC development, yet there are no reported cases in the literature. We present a case of a patient with a history of a previously resected intracranial epidermoid cyst, representing three simultaneous pathologies including a laterally based right frontal NC along with a right corona radiata cavernous malformation lesion, and right middle cerebral artery bifurcation aneurysm. The three lesions were treated microsurgically in one operative session without complications. We discuss the case and review the relevant pathoembryology of laterally based supratentorial NC.

## Introduction

Neurenteric cysts (NCs) are rare congenital developmental lesions of the central nervous system (CNS) that are lined by endodermal-derived epithelium. Several theories have been postulated to explain their incidence but the exact mechanism is not understood and is complicated by the diversity of locations in which NCs appear in the CNS. The neurenteric canal migration hypothesis proposes that endodermal cells migrate through the neurenteric canal, which connects the yolk sac and the amniotic cavity during early gestation, and settles among ectodermal cells prior to neural tube closure [1]. This hypothesis is perhaps the most robust, as it explains the existence of lateral supratentorial lesions as their pathoembryogenesis would not be sufficiently explained by other proposed mechanisms of NC [2]. Furthermore, this mechanism may be supported by past medical history or the coexistence of epidermoid cysts in the CNS, which are thought to arise due to improper neural tube closure potentially increasing the probability of endodermal migration and subsequent NC development; however, there has yet to be any reported incidence in the literature. We present a case of a patient with a previously resected intracranial epidermoid cyst with a laterally based right frontal neurenteric cyst in association with right corona radiata cavernous malformation lesion, and right middle cerebral artery (MCA) bifurcation aneurysm treated microsurgically in one operative session.

## Case presentation

**Figure 1:**
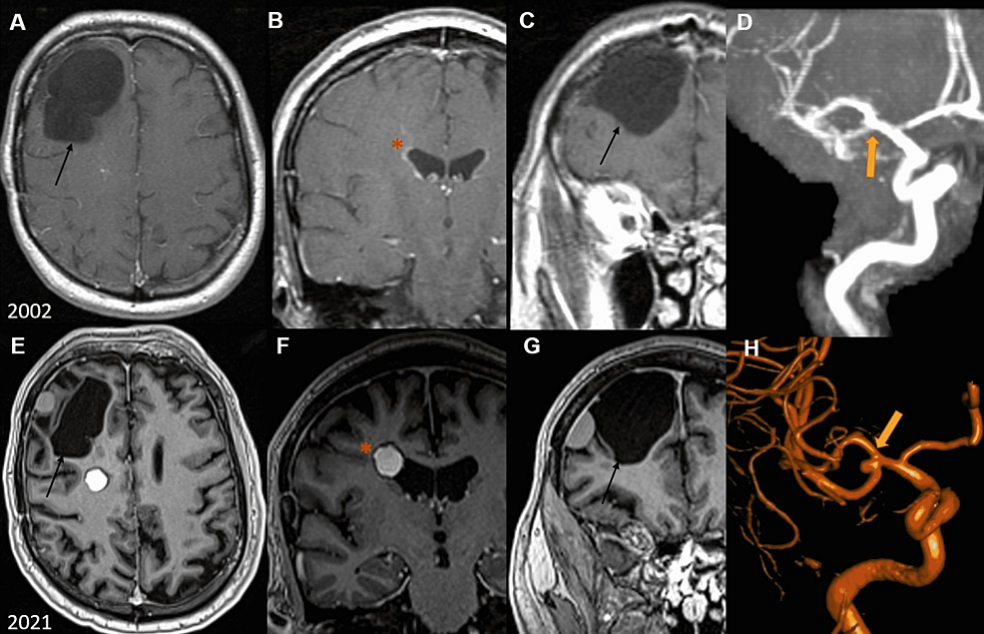
Old images vs. new images Pre-operative MRI from 2002 showing right frontal cystic lesion prior to surgical fenestration (black arrows; A, C), without evidence of cavernous malformation (red asterisk; B) and a smaller middle cerebral artery (MCA) aneurysm (yellow arrow; D). Pre-operative imaging from 2021 showing recurrence of a similarly sized right frontal cystic extra-axial non-enhancing lesion abutting the prior surgical cavity (black arrows; E, G), with a heterogeneous hyperintense mass in the right frontal corona radiata region (red asterisk; F), along with evidence of enlargement of the right MCA bifurcation aneurysm (yellow arrows; D, H).

A 67-year-old male with a past medical history of remote craniotomy for right epidermoid cyst fenestration (~19 years prior with subsequent seizures, been seizure-free since then on anti-epileptics) (Figures 1A-1C) presented to our emergency department (ED) from an outside hospital for evaluation for management of MCA bifurcation aneurysm and right frontal lesions concerning for cavernoma, found on computed tomography angiography (CTA) and magnetic resonance imaging (MRI) obtained for headache and dizziness workup. The patient underwent head CT without contrast showing multiple right frontal hyperdense extra-axial lesions, with one lesion located with the periventricular white matter (Figure 1). Subsequent brain MRI with and without contrast showed a 6.4 x 4.0 x 3.8 cm extra-axial non-enhancing lesion abutting the surgical cavity concerning a large septated surgical bed versus a possible arachnoid cyst (Figures 1E, 1G). Concurrently, a new rounded centrally heterogeneous hyperintense 1.2 x 1.5 x 1.4 cm mass with peripheral hypointensity was noted in the right frontal corona radiata consistent with cerebral cavernous malformation, along with a small developmental venous anomaly located at the posterior border of the lesion (Figures 1E, 1F).

**Figure 2:**
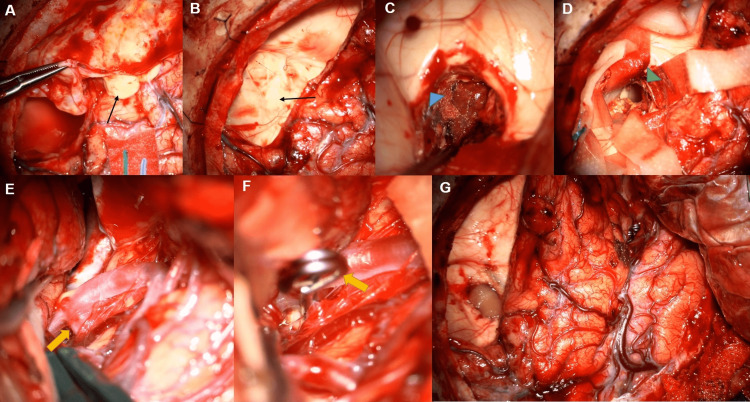
Intraoperative view Intraoperative view of the cyst fenestration draining thick yellowish fluid (black arrows; A, B) and dissection of the cyst wall away from the frontal lobe (blue arrowhead; C). Microsurgical excision of the right corona radiata lesion (green arrowhead; D) and clipping of the right middle cerebral artery bifurcation aneurysm (yellow arrows; E, F) and pre-dural closure view (G).

Subsequently, the patient underwent a diagnostic angiogram, which was concerning for an increase in the size of his aneurysm compared to prior old imaging (obtained during hospitalization), now measuring 4 mm in maximal diameter (Figures 1D, 1H). On clinical examination, the patient was neurologically intact other than headache and dizziness. Following a discussion of the benefits and risks with the patient and his family, the decision was made eventually to proceed with right frontal craniotomy for aneurysm clipping with concurrent resection of the lesions, with intraoperative neuromonitoring (electroencephalogram (EEG), somatosensory evoked potential (SSEP), and transcortical motor evoked potentials (TcMEP)). A bicoronal skin flap was reflected anteriorly to expose the old craniotomy, and a new pterional craniotomy incorporating the inferior portion of the previous craniotomy was performed for adequate exposure. Following fenestration of the cyst draining thick yellowish fluid (Figures 2A, 2B), the cyst wall was dissected away from the frontal lobe (Figures 2C, 2D). This was followed by microsurgical excision of the right corona radiata lesion and clipping of the right MCA bifurcation aneurysm (Figures 2E-2G).

**Figure 3:**
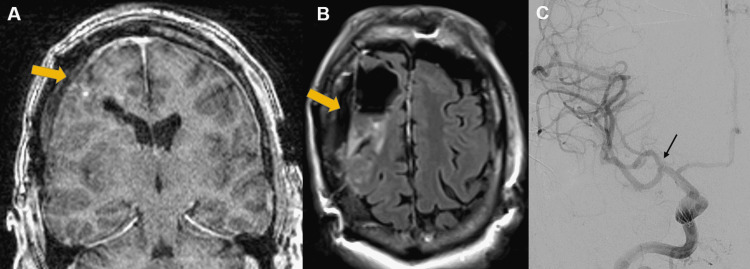
Intraoperative MRI/angiogram images Intraoperative MRI showing gross total resection of the cyst and the cavernous malformation (yellow arrows; A and B), with intraoperative angiogram showing complete exclusion of the aneurysm from the circulation without parent vessel compromise (black arrow; C).

Intraoperative MRI showed gross total resection of the cyst and the cavernous malformation (Figures 3A, 3B), concurrently, complete occlusion of the aneurysm without parent vessel compromise was confirmed on intraoperative catheter angiogram (Figure 3C).

**Figure 4:**
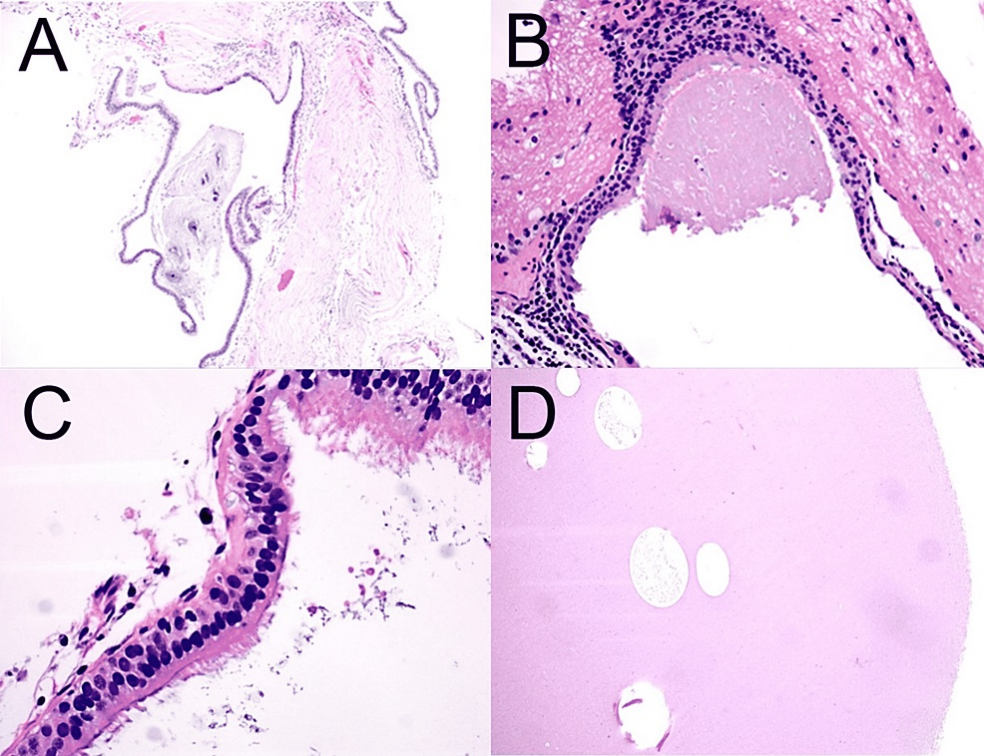
Histologic examination of cyst contents Histologic examination utilizing hematoxylin and eosin staining of cyst linings by ciliated to pseudostratified columnar epithelium and cyst contents (A). Low power view of cyst lining, 5X (B). Low power view of cyst lining and cystic fluid, 10X (C). High power view of cyst lining to demonstrate cilia, 20X, and eosinophilic cyst contents (D).

The final histopathological report confirmed the neurenteric nature of the cyst (Figure 4) with right frontal cerebral cavernous malformations.

The procedure was well tolerated without major complications, other than transient right-sided eye strain that self-resolved within a few days. The patient remained neurologically intact on subsequent clinical follow-up.

## Discussion

This rare case describes the appearance of a supratentorial neurenteric cyst (SNC) in addition to a newly formed cavernous malformation and an MCA aneurysm, which were all treated in one surgical procedure. It remains unclear why this patient had three different pathologies in close proximity and the patient did not receive radiosurgery after his initial surgery 20 years ago excluding a radiosurgery-induced cavernoma. The extreme rarity of SNC renders them unlikely contenders on the list of differentials for frontal cysts [3]. NCs are more commonly found in the intradural extramedullary ventral aspect of the spinal cord [3], and intracranial NCs are typically located in the posterior fossa, fourth ventricle, or close to the brainstem [3-5]. Moreover, SNCs have variable-reported radiographic properties as their MRI signal intensity varies based on the cyst protein content [6]. They are usually isointense to slightly hypointense on T1-weighted sequences and hyperintense on T2-weighted/fluid attenuation inversion recovery (FLAIR) sequences [5,7]. In our case, the lack of enhancement and diffusion restriction averted meningiomas and epidermoid cyst from the differential probabilities, yet the lesion was isointense to cerebrospinal fluid (CSF) (Figures 2E, 2F), shifting the preoperative diagnostic possibilities to postoperative cystic surgical cavity versus arachnoid cyst. The exact mechanism behind these lesions’ formation is yet to be elucidated, and several theories have been proposed to explain the presence of endodermal-derived epithelium in the CNS. The aberrant incorporation of primitive endodermal cells in the notochord secondary to separation failure of the notochord from the foregut has been postulated as an underlying mechanism [8]; however, this does not explain the supratentorial lesions given that the rostral endodermal level terminates at the clivus [6,8]. Similarly, the “Seessel's pouch origin” hypothesis of a common origin of SNC with colloid and Rathke's cleft cysts would not explain the laterally located lesions [2,6]. A third theory proposes that endodermal cells migrate through the neurenteric canal into the amniotic cavity where they could come to settle among the ectodermal cells.

**Figure 5:**
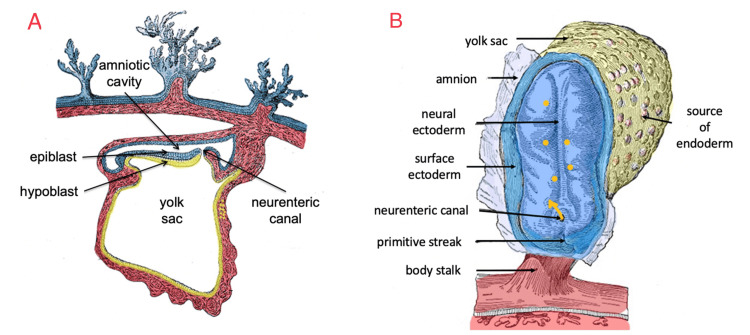
Pathoembryological hypothesis Adapted figures illustrating the neurenteric canal migration hypothesis. Image (A) shows a sagittal cut of a two-week embryo illustrating how the neurenteric canal connects the amniotic cavity with the yolk sac. Image (B) shows a dorsal view of the embryonic disc of a three-week embryo illustrating how endoderm cells could be dispersed via the neurenteric canal into the amniotic cavity to settle in various locations in the CNS. The figure uses images that were published in the 1918 edition of Gray's Anatomy, which was originally authored by Henry Gray (1827-1861). According to copyright terms, it is in the public domain in the country of publication (United States) and any other countries where the copyright term is the author’s life plus 100 years. Retrieved May 18, 2022, from Gray, H. (1918). Anatomy of the Human Body (W. L. Lewis, Ed.; 20th ed.), Lea & Febiger [10].

The neurenteric canal migration hypothesis would be able to explain the diversity of neurenteric locations, including lateral supratentorial lesions as in our case. In early gestation, the neurenteric canal connects the amniotic cavity and yolk sac and is thought to equalize the hydrostatic pressure between the fluid filling the primitive amniotic cavity and that filling the yolk sac (Figure 5) [9]. Without this, the integrity of the bilaminar embryonic disc could potentially be compromised [9]. As gastrulation begins, epiblast cells migrate into the primitive node and displace hypoblast cells to form the endoderm. At this stage, it is possible that endodermal cells could traverse the neurenteric canal into the amniotic cavity and settle among the ectodermal cells that will subsequently form the neural tube (Figure 5). This may explain the various locations of NC, in particular, the most frequent locations, especially if this dispersal occurred when the neural groove was still open.

Our patient’s previous intracranial epidermoid cyst is consistent with the neurenteric migration hypothesis and may help further elucidate SNC pathogenesis. Epidermoid cysts are benign congenital lesions that are thought to arise when ectodermal cells become entrapped in the neural tube due to improper closure [11]. While the two different cysts arise from two different germ layers, it is plausible that the pathogenesis of SNC can also be attributed to improper closure of the neural tube, perhaps delayed, which would increase the likelihood of an endodermal cell being displaced by the neurenteric canal migration hypothesis to the far supratentorial and lateral regions. This case report potentially helps to elucidate our current understanding of SNC embryological pathogenesis and may highlight the importance of raising them in the list of differentials for patients with a past medical history of epidermoid cysts located in the CNS.

However, there has yet to be any reported case of concurrent intracranial epidermoid cysts. Co-existence of an intraparenchymal subependymoma was reported in one case of SNC [12]. Furthermore, there have not been any reported cases of a coexistent cavernous malformation lesion and right MCA bifurcation aneurysm, which were treated along with the SNC in one operative session. The pathophysiological underpinnings of the concurrent cavernous malformation lesion and the MCA aneurysm, in this case, are unknown; nonetheless, this case provides some insight into embryological pathogenesis of these laterally located cysts along with reporting unusual simultaneous co-occurrence of these three pathologically distinct lesions.

## Conclusions

We report a rare case of a lateral SNC in close proximity to a newly formed cavernous malformation and an MCA aneurysm, all of which were surgically treated in one procedure. The embryological mechanism of pathogenesis that leads to the development of lateral SNC is yet to be fully elucidated. The neurenteric canal migration hypothesis might be a reasonable explanation of their pathogenesis. It is also plausible that improper neural tube closure, which is thought to contribute to epidermoid cysts development in the CNS, likewise contributes to SNC pathogenesis. Future studies to investigate the relationship between CNS epidermoid cysts and SNC are warranted.
